# Hydriodate of Potash in Acute Hydrocephalus

**Published:** 1847-08

**Authors:** 


### ARTICLE II.

### HYDRIODATE OF POTASH IN ACUTE HYDROCEPHALUS.

We have received a letter on the use of Hydriodate of Potash in Acute Hydrocephalus, from P. A. Allaire, M.D.

Dr. Allaire, is not a believer in the efficacy of this medicine and supports his views by showing the inconclusiveness of the cases cited in its favor. In addition to this method, by which he very successfully shows that the evidence thus far adduced in its favor is not sufficient to establish its claims, our own experience has furnished us with two well-marked cases in which it entirely failed to produce the slightest effect. It is a disease of which the symptoms are often similated by gastric and intestinal irritation and prostration; and in this region, very often by "miasma," or the unknown cause of intermittent and remittent fever. Of this kind Dr. Allaire thinks were the cases of Dr. Thompson Mead, reported in the last number of our Journal, and he attributes their cure to the judicious treatment employed in addition to the Hyd. Potass. D. B.

